# Identifying common impairments in frail and dependent older people: validation of the COPE assessment for non-specialised health workers in low resource primary health care settings

**DOI:** 10.1186/s12877-015-0121-1

**Published:** 2015-10-14

**Authors:** Jotheeswaran AT, Amit Dias, Ian Philp, John Beard, Vikram Patel, Martin Prince

**Affiliations:** Department of Ageing and Life Course, World Health Organization, Geneva, Switzerland; Public Health Foundation of India, New Delhi, India; Hull and East Yorkshire Hospitals NHS Trust, Hull, UK; Department of Preventive and Social Medicine, Goa Medical College, Goa, India; Sangath, Goa, India; Centre for Global Mental Health, London School of Hygiene and Tropical Medicine, London, UK; Department of Ageing and Life Course, World Health Organization, Geneva, Switzerland

**Keywords:** Geriatric assessment, Frailty assessment, Frail older people, Dependence, Ageing, Case-finding, Primary health care settings, India

## Abstract

**Background:**

Frail and dependent older people in resource-poor settings are poorly served by health systems that lack outreach capacity. The COPE (Caring for Older PEople) multidimensional assessment tool is designed to help community health workers (CHWs) identify clinically significant impairments and deliver evidence-based interventions

**Methods:**

Older people (*n* = 150) identified by CHWs as frail or dependent, were assessed at home by the CHW using the structured COPE assessment tool, generating information on impairments in nutrition, mobility, vision, hearing, continence, cognition, mood and behaviour. The older people were reassessed by local physicians who reached a clinical judgment regarding the presence or absence of the same impairments based upon clinical examination guided by the EASY-Care assessment tool.

**Results:**

The COPE tool was considered easy to administer, and gave CHWs a sense of empowerment to understand and act upon the needs of older people. Agreement between COPE assessment by CHW and clinician assessors was modest (ranged from 45.8 to 91.3 %) for most impairments. However, the prevalence of impairments was generally higher according to clinicians, particularly for visual impairment (98.7 vs 45.8 %), cognitive impairment (78.4 vs. 38.2 %) and depression (82.0 vs. 59.9 %). Most cases identified by WHO-COPE were clinician confirmed (positive predictive values - 72.2 to 98.5 %), and levels of disability and needs for care among those identified by COPE were higher than those additionally identified by the clinician alone.

**Conclusions:**

The COPE is a feasible tool for the identification of specific impairments in frail dependent older people in the community. Those identified are likely to be confirmed as having clinically relevant problems by clinicians working in the same service, and the COPE may be particularly effective at targeting attention upon those with the most substantial unmet needs.

**Electronic supplementary material:**

The online version of this article (doi:10.1186/s12877-015-0121-1) contains supplementary material, which is available to authorized users.

## Background

Among older people, impairments in mobility, nutrition, vision, hearing, cognition, mood, and behaviour make an important contribution to years lived with disability, and dependence, and mortality [1–4]. A recent systematic review concluded that, in primary care and community settings, interventions targeting risk factors and functional impairments may be more effective than disease specific interventions at alleviating burden in older people with complex multimorbidity [5]. Such an approach may be particularly salient to low and middle-income countries with few physicians, where non-specialist community health workers could be used to improve the coverage of and access to health and social care. However, identifying impairments that limit older people’s functional capacity, and selecting and implementing evidence-based interventions will be a significant challenge in such settings.

In India, as with many other low and middle-income countries (LAMIC), the primary health care system is the core of the government’s provision for basic health care needs. However, studies conducted in LAMIC indicate highly variable levels of utilization of government primary health care services among older people, with a preference for private doctor and hospital outpatient services in India and some Latin American countries [6]. The public health care system is acknowledged to be limited, in that it is mostly clinic-based with little or no outreach, focuses upon the detection and treatment of acute illnesses, and fails to provide coordinated continuing care to those with chronic conditions [7, 8]. In India, it is not considered part of the primary health care physician’s role to make home visits to assess and treat those who cannot access health facilities [9]. A cadre of Auxiliary Nurse Midwives (ANM), often referred to as community health workers (CHWs) was introduced 50 years ago to increase the coverage of basic health care at the community level, and improve equity [10]. CHWs undergo nine or 24 months training focused mainly upon midwifery and maternal and child health. Their role is to supplement that of doctors and other higher trained personnel by promoting preventive and curative health activities. CHWs have become key workers at the interface of primary health care services and the community, and their effectiveness is reflected in secular reductions in maternal and child mortality [11]. Only recently has interest shifted to the potential to engage CHWs in the task of controlling chronic non-communicable diseases [12]. As the only branch of primary care offering outreach into the community, and with a family and household orientation to their work, CHWs are in principle ideally situated to implement age-appropriate care for older people; case-finding (identifying frail or dependent older people in the community, who have not sought help at the health facility), and home-based assessment and intervention to treat or mitigate the effects of impairments arising from chronic disease.

We have already demonstrated that, after 3 h training, CHWs working in the Goa State health service could accurately identify frail, dependent, or frail and dependent older people [13]. The next step was to develop and evaluate a simple structured assessment that would enable CHWs to identify specific impairments at the level that could inform targeted evidence-based intervention. In support of this approach, a review of studies conducted in high income countries concluded that home visits based on comprehensive geriatric assessment can reduce functional decline in older people [14]. Little research has been undertaken to validate the assessments that could be used by non-specialised community health workers in resource-poor settings [15, 16].

The scope of the assessment was determined by a concurrent World Health Organization program (WHO-COPE) to develop evidence-based guidelines for the prevention and management of dependence by non-specialist health workers; covering nutrition, mobility, falls, cognition, mood and behaviour, sensory impairment, and incontinence [17]. It was assumed that CHWs would lack prior experience in assessing older people, and hence structured assessments with objective tests would be required, rather than the exercise of clinical judgment. Scoping the literature failed to identify any comprehensive multi-dimensional assessment that was simple, fully structured and capable of identifying and distinguishing between specific impairments. Existing comprehensive assessment tools recommended for use in older people [18–21] were either too generic, or required specialised clinical knowledge for administration and interpretation.

The aims of the current study were therefore to develop a comprehensive assessment tool for CHWs working in the primary health care system, to assess the feasibility and acceptability of this approach, and to explore concurrent validity against clinical assessments carried out by physicians working in the same local public health system. It would not be appropriate to consider such assessments as a ‘gold standard’ criterion, since these doctors were non-specialists, and lacked the time or equipment for a rigorous comprehensive clinical examination. The approach was, rather, to assess pragmatically whether those identified by the CHWs would be likely to be confirmed as requiring intervention by local clinicians

## Method

### Design

Older people identified by CHWs as frail or dependent, were assessed at home by the CHW using the COPE assessment tool (see below for details), generating information on impairments in nutrition, mobility, vision, hearing, continence, cognition, mood and behaviour. After an interval of up to 3 weeks, they were re-assessed by local physicians who reached a clinical judgment regarding the presence or absence of the same impairments based upon clinical examination guided by the EASY-Care assessment tool [22]. This study was conducted between 2013 and 2014. King’s College Research Ethics Committee and Institutional Ethics Committee of Public Health Foundation of India approved the study.

### Development and structure of COPE assessment

Rapid review was conducted to select appropriate assessments for undernutrition, mobility and strength impairments, visual and hearing impairments, and cognition, mood and behavioural impairments. The selection of measurements was based on the following criteria: a) they should be simple, quick, and easy to administer in a primary health care facility, or the older person’s own home, b) they should be capable of being administered by non-specialist health workers with suitable training, c) they should have good sensitivity, specificity and positive predictive value for identification of the target impairment. The full COPE assessment tool comprises; Section 1 - demographic information; Section 2 - assessments for specific impairments; Section 3 - a brief interview with co-resident or primary caregiver; Section 4 - a summary of findings and action plans for management or referral. The time taken to administer the full COPE assessment ranges from 30 to 45 min. The following description focuses upon Section 2, the assessment of impairments.

- Assessments of mobility: A 10 m walk test, and the chair stand test were used to identify mobility impairment. Both are well-suited to standardised evaluation of older people at community level by non-specialist health workers, being quick to administer, inexpensive, and a reliable measure of frailty with respect to physical functioning [23–26]. The walking test, used successfully in the 10/66 Dementia Research Group population-based studies in LAMICs, involves the participant being timed walking 5 m (indicated by a piece of string), turning and returning to the starting point; with time taken to turn taken into account, a cut off of more than 15 s to complete the test was considered to reflect limited mobility (<1.2 m per second) [27, 28]. Although often considered to be a good proxy measure of sarcopaenia (loss of muscle mass and strength) gait speed will also reflect impairments in the function of joints, central and peripheral nervous system. The ‘30 s chair stand’ test assesses proximal lower limb strength, and has also been used in LAMICs [29]. The person being assessed is asked to stand upright from a chair with their arms folded across their chest, then to sit down again and then to repeat the action at their own pace. The test score is the number of times they rise to a full stand from the seated position within 30 s. A cut-off of fewer than seven stands in 30 s was recommended for detecting older people with, or at risk of, lower limb strength impairment [30]. Fewer than 14 stands predicted falls in a study conducted among community-dwelling older people in Japan [31]. Performance may be influenced by the height of the chair, leading to problems with standardisation when used in the community. Also, a high proportion of frail participants may be unable to perform the task, leading to floor effects.
- Assessment of nutritional status: The mini-nutritional assessment (MNA-SF®) is a short form version of the original 18 item MNA full version, comprising six items that best discriminated between malnourished, at risk, and normal older people [32, 33]; decline in food intake; weight loss in the last three months; mobility limitation; psychological stress or acute diseases in the past three months; neurological problems (dementia and depression); and body mass index (BMI). BMI, requiring accurate assessment of height and weight is difficult to measure in the community, particularly in bed- or chair-bound older people. In the revised MNA, calf-circumference was substituted for BMI, with good criterion [34] and predictive validity [35]. MNA-SF has a maximum score of 14 points, with risk of malnutrition increasing with lower scores. Respondents are classified as well-nourished (a score of 12–14), at-risk for malnutrition (8–11), or malnourished (0–7).
- Visual impairment: The Snellen ‘tumbling E’ chart has been used in population-based studies to identify visual impairment in older people in India [36]. Although developed for use in children, it has a general application for low literacy groups, and has been used and validated in many developing countries, including among older people [37–39]. According to the World Health Organisation, visual impairment is defined as a best-corrected visual acuity of less than 6/18 in the better-seeing eye [40].
- Hearing impairment: The whisper voice test [41, 42] was administered to identify hearing impairment. The examiner stands behind the seated older person and enunciates three random numbers (for example, 2-6-9) at four decreasing levels of loudness: a conversational voice at 6 inches and 2 ft from the ear and then a whispered voice at the same distances. Tests were presented to each ear, masking the other by rubbing the tragus. If correct, the examiner proceeds to the next level of difficulty, if incorrect, the test is repeated using different numbers. A pass at each level is achieved if the three numbers are repeated correctly or if at least three out of six numbers are repeated correctly over two sets [43]. Failing the whisper voice test at 2 ft implies a 30 dB hearing loss, likely to have a significant impact on communication. Sensitivity and specificity against audiometry ranges from 90 to 100 % and 80 to 87 % respectively [44], with little difference when administered by experienced and inexperienced examiners [45]. However, studies validating the whisper voice test were conducted in hospital or institutional settings, exclusively in high income countries [44].
- Cognitive impairment: The Community Screening Instrument for Dementia (CSI-D) was extensively validated against clinician dementia diagnosis (DSM-IV dementia) in 26 centres in Latin America, India and SE Asia [46]. It combines culture and education-fair cognitive testing of the participant and an informant interview enquiring after the participant’s daily functioning and general health, into a single predictive algorithm. The Brief version of CSI-D (administered in around 5 min) was developed using item response theory for item reduction, the intention being to make the assessment brief enough to be used as a screening assessment by non-specialist health workers in low resource primary care settings [47]. The brief version comprises seven cognitive test items for the older person and six informant report items for a co-resident or primary caregiver. Lower scores in the cognitive test and higher scores in the informant reports indicate cognitive impairment. To calculate the total score, the informant score is subtracted from the cognitive score, giving a possible range of −6 to +9 with a cut-off of less than five reported to have 97.3 % sensitivity and 90.5 % specificity in detecting older people with dementia, based on data from community surveys [47]. It has not previously been used by non-specialist health workers in low resource settings.
- Mood: The Geriatric Depression Scale (GDS) was originally devised with 30 items specifically for use in older populations [48], and has been used successfully in an illiterate older Indian population [49]. A 15 item short version is more widely used, but is still time-consuming to administer. An eight-item version, the GDS-8 has been developed in the Netherlands for brevity and ease of use in nursing home residents. The GDS-8 is internally consistent (alpha = 0.80) and against clinician interviews yielded a sensitivity of 96.3 % for major depression and 83 % for minor depression with specificity of 71.7 % at a cut-off point of 2/3 [50]. The GDS-8 item short version has not been validated in community settings.
- Behaviour: The brief form of the Neuropsychiatric Inventory (NPI-Q) comprises 12 questions administered to an informant, covering common behavioural and psychological symptoms: delusions, hallucinations, agitation/aggression, depression, anxiety, elation/euphoria, apathy, disinhibition, irritability/lability, aberrant motor activity, sleep and night time behaviours, appetite change and eating behaviour [51]. Each behaviour or symptom is rated by the informant on a six point scale (0–5) for the distress it occasions them. The total NPI-Q distress score is the sum of the 12 individual domain scores, with a maximum possible score of 60. NPI-Q has adequate test-retest and inter-rater reliability as well as good concurrent validity [52]. A behavioural problem was considered as significant, only if it was rated by the caregiver as causing distress.
- Dependence: Care dependency (whether the participant needed no care, some care or much care) was ascertained through a series of open-ended questions administered to the informant.

### Training CHWs to use the COPE assessment

Training was conducted by two facilitators for ten CHWs currently working in Sub-Health Centres of Corlim Primary Health Centre, Goa, India. Training involved a) brief introduction to common problems associated with ageing, age-dependent chronic diseases, and the origins and types of needs for care arising in frail and/or dependent older people, b) a detailed description of each impairment and the relevant COPE assessment methods, and c) general rules for identifying impairments, emphasizing the use of the specified test cutpoints, but also recommending procedures for exercising judgment when the relevant test was difficult or impossible to administer. Facilitators demonstrated the correct method of performing the assessment in an older person’s home. Safety precautions were clearly flagged. After each demonstration, the facilitators invited CHWs to demonstrate how they would perform the assessment with the facilitator acting as the older person. Other trainees observed the role-play and commented on their colleague’s performance. The facilitator summarised the background knowledge and required competencies, and health workers were given the opportunity to clarify doubts. Facilitators asked questions to check that trainees had understood the assessment procedure and general rules for identifying impairments. Finally, each CHW was requested to identify an older person for whom they believed a COPE assessment would be indicated. These assessments were observed by the facilitator who noted any deviations from assessment protocol, which were then fed back to the CHW. Any doubts or questions raised by the CHW were also clarified at this stage.

### Clinician assessment

Clinician assessments were guided by the EASY-Care Standard (2010) assessment [53] comprising scales and single items derived from established instruments, including the Barthel index [54], the Duke OARS IADL scale [19], the SF-36 [55], questions on cognitive function [56], the four-item geriatric depression scale [57], and questions from the World Health Organisation 11 countries social and medical survey instrument [58]. The EASY-Care assessment, as a package, had shown content, discriminant, and cross-cultural validity [59–61]. Forty-nine checklist items are clustered into seven groups; seeing, hearing and communicating; looking after yourself; getting around; your safety; your accommodation and finance; staying healthy; and your mental health and wellbeing. The assessment can be used by a suitably trained clinician to identify and prioritise management of unmet needs. Based on 18 ADL and IADL items, EASY-Care also generates summary scores for ‘independence’ (higher scores indicating needs for care and support), risk of breakdown in care, and risk of falls. We have demonstrated that the independence score scale has excellent core psychometric properties, with high internal consistency, and strong hierarchical scale properties [13]. Although the 49 checklist items are structured and quite well operationalised, identification of unmet needs and development of management plans requires the exercise of clinical judgment, hence the choice of this assessment for the clinician validation rather than the CHW assessment. EASY-Care assessment was supplemented by clinical assessments routinely used by primary care doctors (see Table 1). Three doctors with minimum ten years of primary care experience were included. Two of the medical doctors had completed their clinical training in psychiatry and neurology. Clinical judgment was then applied, based upon the entirety of available evidence to identify impairments in nutrition, mobility, vision, hearing, continence, cognition, mood and behaviour.

**Table 1:** COPE assessment and criteria, and clinician assessment for the identification of impairments

Impairments	COPE	COPE criterion	Clinical examination^a^
Nutrition	Mini nutritional assessment (MNA-SF®)	‘Malnourished’ (MNA score of <8)	Muscle bulk. Diet history. History of health conditions related to undernutrition. Current weight and history of weight loss. Oral and dental health.
Mobility	10 m walking test	Complete the walking test in > 15 s, and/or <7 chair stands in 30 s, or could not participate in the tasks because of severely restricted mobility.	Neurological examination, including power in major muscle groups. ADL difficulties.
	Chair-stand test		EASY-Care checklist: Can you move yourself from bed to chair? Can you get around indoors? Can you manage stairs? Can you walk outside?
Vision	Snellen ‘tumbling E’ visual acuity chart	Visual acuity <6/18 in one or both eyes, or CHW impression of visual impairment for those not able to complete test	Counting fingers, hand motion, light perception.
			EASY-Care checklist: Can you see (with glasses if worn?)
Hearing	Whisper voice test	Failed whisper voice test at 2 ft	Weber and Rinne tests. Vestibular function.
			EASY-Care checklist: Can you hear (with hearing aid if worn)?
Continence	Single item from informant CSI-D ‘Does she have difficulty using the toilet? Does she wet of soil herself?’	Coded	EASY-Care checklist: Do you have accidents with your bladder? Do you have accidents with your bowels?
		0. No problems	
		1. Occasionally wets bed	
		2. Frequently wets bed	
		3. Double incontinence	
Cognition	Brief Community Screening Instrument for Dementia (CSI-D)	Combined score of <5	CNS Higher Functions; mental status examination; family history, medical history (underlying mental health conditions), addictions.
			EASY-Care checklist: Do you have any concerns about memory loss or forgetfulness? Do you feel lonely? Have you suffered from any recent loss or bereavement?
			In the past month…
			Have you had any trouble sleeping? Have you had bodily pain? Have you often been bothered by feeling down, depressed or hopeless? Have you often been bothered by having little interest or pleasure in doing things?
Mood	Eight item Geriatric Depression Scale (GDS-8)	GDS score of > =3, or (for those not able to respond), informant report of depressed mood (NPI-Q q.4)	
Behaviour	12 item Neuropsychiatric Inventory (NPI-Q)	One or more behavioural or psychological symptoms causing caregiver at least some distress	

^a^For clinician assessment, the criterion was ‘clinical judgment’ in all cases

### Qualitative interview with CHWs

The purpose of the qualitative study was to elicit information regarding CHW’s experiences and opinions about the administration of the COPE structured assessment, to clarify its potential for routine primary health care practice in the community. A research assistant trained in qualitative interviewing conducted individual in-depth interviews with ten CHWs who had administered the COPE assessment for frail dependent older people, and had provided informed consent to participate in the qualitative study. All qualitative interviews were conducted in sub-health centres, and the duration of each interview ranged between 45 and 90 mins. Interviews were mainly conducted in Konkani (Goan local language). All interviews were recorded, transcribed and translated in to English before thematic analysis was carried out.

## Data analysis

The proportion of older people considered, according to the CHW COPE assessments, to have impairments in nutrition, mobility, vision, hearing, continence, cognition, mood and behaviour, was described, and the independent effects of age (per year) and gender (male versus female) assessed using Poisson regression to generate prevalence ratios. The prevalence of each impairment according to CHW COPE assessment was compared with that from clinician judgment. The agreement between CHW assessment and clinician judgment was assessed using the % of overall agreement, and Cohen’s kappa. The sensitivity, specificity, positive and negative predictive values of the CHW assessment were estimated using clinician judgment as the external reference criterion.

The construct (concurrent) validity of the CHW COPE assessments was assessed by:

1) comparing mean EASY-Care independence scores for those identified as impaired in the CHW COPE assessment (‘true positives’ and ‘false positives’ combined) with those who were identified as impaired only according to clinician judgment (‘false negatives’) and those identified as impaired according to neither criteria (‘true negatives’), using one way ANOVA and Scheffe tests for statistical significance accounting for multiple sub-group comparisons; 2) assessing the correlations between number of impairments identified by CHW COPE assessment, needs for care assessed by CHW, numbers of impairments identified by clinician judgment, and EASY-Care independence scores; and 3) using multiple linear regression to assess the independent individual and collective contribution of a) CHW identified impairments and b) clinician identified impairments to the percentage of variance in EASY-Care independence scores, having controlled for age and gender.

Two researchers independently analysed the interview transcripts. A grounded theory method was used for content coding and identification of themes. Data analysis was performed at three stages. First, two researchers independently marked the key text with a series of codes emerging from the transcripts. Secondly, codes were grouped together as representing similar concepts. Third, identified codes reflecting similar concepts were classified under broad themes for better understanding and description of findings. NVivo software version 8 was used for data analysis.

## Results

The ten trained community health workers (CHWs) referred the sub-health centre case registry to identify people aged 60 years and over, and reviewed the family record, and their own recollections of family visits to identify those who, on the basis of their training, could be considered to be frail and/or dependent. The 159 so identified were approached by the CHWs for informed consent. Seven refused to participate and two others completed the COPE assessment but could not participate in the clinician assessment due to hospitalisation. The COPE assessment was administered by CHWs at the older person’s home. Subsequently, the clinician made a home visit to conduct the clinical examination; however, for three frail dependent older people the clinical examination was performed at the primary health care facility. The mean age of the participants was 73.6 years (SD 7.6). Most were women (72 %), and 118 (78 %) had no education. Forty-eight (31 %) were still married and 102 (67 %) widowed. Only four (3 %) were living alone, while 121 (80 %) lived with children and/or children-in-law. Nine (6 %) were in paid full- or part-time employment. The very large majority, 141 (94 %), indicated that they did receive care and support, 12 of whom also reported providing care for someone else.

### Distribution of impairments as assessed by the CHW using the COPE assessment

Valid data was obtained for most participants for most of the assessments. However, mainly because of severe mobility impairment, 27 (17.8 %) could not attempt the chair-stand test, and 21 (13.8 %) could not attempt the walking test. Mainly because of cognitive impairment, 19 (12.7 %) could not be tested for visual acuity, eight (5.3 %) could not be assessed for hearing, and 12 (7.9 %) could not provide meaningful responses for the Geriatric Depression Scale. According to data collected by the CHWs in their structured assessments, the most common impairment was mobility (*n* = 124, 81.6 %), followed by hearing (104, 68.4 %), mood (91, 60.7 %), nutrition (82, 53.9 %), behaviour (73, 48.0 %), vision (66, 45.8 %), cognition (58, 38.2 %) and continence (34, 22.4 %). Of the 56 identified by CHWs as having visual impairment, 16 were considered to have refractable errors (corrected by pinhole) and 40 unrefractable errors. Among those identified with incontinence, 21 had occasional urinary incontinence, six frequent urinary incontinence, and seven faecal incontinence. The commonest behavioural disturbances reported to be distressing by the carer were appetite and eating problems (*n* = 29), agitation or aggression (*n* = 25), sleep disturbance (*n* = 23), depression (*n* = 23), irritability (*n* = 20), and apathy (*n* = 19).

**Fig. 1:**
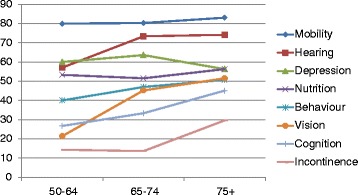
Prevalence of impairments (as identified by the community health worker, using the COPE assessment), by age

Only two older people were rated as having no impairments; 28 (18.4 %) had one or two impairments, 55 (38.2 %) had three or four impairments, and 67 (44.1 %) had five or more. Most (82.8 %) were assessed as having needs for care; 72 (47.4 %) were identified as needing occasional care, and 54 (35.5 %) as needing care much of the time. The independent effects of age and gender on the prevalence of the impairments are described in Table 2 and (age only) in Fig. 1. While the prevalence of all impairments other than depression, mobility and undernutrition increased monotonically with increasing age, only the effect of age on the prevalence of cognitive impairment was statistically significant in this group of older people identified as having frailty and/or needs for care. There were no differences in the prevalence of any impairments with gender.

**Table 2:** Independent effects (Prevalence Ratios) of age and sex on the prevalence of common impairments, as assessed by the community health worker

Impairment	Effect of age (in years, controlling for sex)	Effect of sex (men compared with women, controlling for age)
Nutrition impairment	1.01 (0.98–1.04)	1.11 (0.68–1.83)
Mobility impairment	1.00 (0.96–1.03)	0.87 (0.49–1.57)
Vision impairment	1.02 (0.99–1.05)	0.91 (0.54–1.55)
Hearing impairment	1.01 (0.99–1.04)	1.41 (0.88–2.28)
Incontinence	1.05 (1.00–1.10)	0.58 (0.29–1.17)
Cognitive impairment	1.04 (1.01–1.07)	1.39 (0.76–2.56)
Depression	0.99 (0.97–1.02)	1.01 (0.63–1.62)
Behavioural impairment	1.01 (0.99–1.04)	1.27 (0.74–2.17)

### Agreement between CHWs COPE assessment and clinician diagnosis

The agreement between the CHW identification of impairment, and the rating of the clinician assessor is summarised in Table 3. The agreement (kappa) was generally modest; between 0.20 and 0.41 for undernutrition, incontinence, depression and impairments in hearing and behaviour, 0.14 for mobility, 0.12 for cognitive impairment, and −0.02 for vision impairment. Other than hearing impairment, the prevalence of each impairment was always higher according to the judgment of the clinician. While overall agreement proportions were generally high, ‘false negatives’ (clinician +/ CHW -) were more numerous than ‘false positives’ (clinician -/ CHW +). This was particularly striking for visual impairment, where according to the clinicians 98.7 % were impaired but according to the CHW assessment only 45.8 %, cognitive impairment (78.4 versus 38.2 %) and depression (82.0 versus 59.9 %). The overall pattern comparing CHW assessment with the assumed ‘gold standard’ of clinician assessment was therefore one of moderate sensitivity and specificity for each of the assessments, with a generally high positive predictive value (exceeding 78.0 % for all assessments), and low negative predictive value. For those CHW administered COPE assessments that generated continuously distributed scores, the area under the ROC curve (AUROC) suggested only moderate discriminability with respect to the relevant impairment according to clinician judgment with AUROC close to 0.70 for most tests, but somewhat lower for mobility (walking test, AUROC 0.63; chair stand test, AUROC 0.65).

**Table 3:** Validity of COPE community health workers assessment against clinical diagnosis as external reference criterion

	Prevalence according to COPE assessment and clinician		Agreement between COPE assessment and clinician (CHW first)				Indicators of agreement		Validity coefficients			
Impairments	CHW/ COPE	Clinician judgment	+/+	+/−	−/+	−/−	% Overall agreement	Kappa value (SE)	Sensitivity % (95 % CI)	Specificity % (95 % CI)	Positive predictive value %	Negative predictive value %
Nutrition	82 (53.9 %)	98 (65.3 %)	64	18	34	34	65.3 %	0.28	65.3 %	65.4 %	78.0 %	50.0 %
		MV = 2						(0.07)	(55.0–74.6 %)	(50.9–78.0 %)	(67.5–86.4 %)	(37.6–62.4 %)
Mobility	124 (81.6 %)	137 (91.3 %)	113	8	24	5	91.3 %	0.14	83.2 %	38.5 %	93.4 %	17.9 %
		MV = 2						(0.07)	(75.8–89.0 %)	(13.8–68.4 %)	(87.5–97.1 %)	(6.1–36.8 %)
Vision	66 (45.8 %)	148 (98.7 %)	64	1	76	1	45.8 %	−0.02	45.7 %	50.0 %	98.5 %	1.3 %
	MV = 8	MV = 2						(0.02)	(33.2–50.8 %)	(1.3–98.7 %)	(90.3–99.9 %)	(0.03–7.1 %)
Hearing	104 (68.4 %)	99 (66.0 %)	77	26	15	24	71.1 %	0.33	83.7 %	48.0 %	74.7 %	61.5 %
	MV = 8	MV = 2						(0.08)	(74.5–90.6 %)	(33.6–62.6 %)	(65.2–82.8 %)	(44.6–76.6 %)
Continence	34 (22.4 %)	53 (34.9 %)	25	8	29	88	75.2 %	0.41	47.2 %	90.7 %	73.5 %	75.9 %
	MV = 1	MV = 2						(0.08)	(33.9–60.5 %)	(84.9–96.5 %)	(58.4–68.6 %)	(68.1–83.7 %)
Cognition	58 (38.2 %)	116 (78.4 %)	49	7	67	25	50.0 %	0.12	42.2 %	78.1 %	87.5 %	27.2 %
		MV = 4						(0.05)	(33.1–51.7 %)	(60.0–90.7 %)	(75.9–94.8 %)	(18.4–37.4 %)
Mood	91 (59.9 %)	123 (82.0 %)	81	10	42	17	65.3 %	0.20	65.9 %	63.0 %	89.0 %	28.8 %
		MV = 2						(0.07)	(57.4–75.5 %)	(42.4–80.6 %)	(79.4–94.2 %)	(19.5–45.5 %)
Behaviour	73 (48.0 %)	84 (56.0 %)	52	20	32	46	65.3 %	0.31	61.9 %	69.7 %	72.2 %	59.0 %
								(0.08)	(66.9–85.8 %)	(47.8–72.4 %)	(61.0–80.4 %)	(54.3–79.4 %)

### Concurrent validity of COPE assessment

The continuously distributed COPE assessments for impairment in cognition, nutrition, mobility and behaviour correlated statistically significantly, but moderately (correlation coefficient 0.21 to 0.47) with the EASY-Care independence score (Table 4). Correlations of the GDS-8 depression score and the number of hearing tests passed (from 0 to 4) in the best or worst ear with EASY-Care independence score, were negligible or low, and not statistically significant. The numbers of impairments identified by the CHW-administered COPE assessment and by clinician judgment correlated moderately (Kendall’s Tau-B +0.38, *p* < 0.001). The Kendall’s Tau-B correlation between numbers of impairments according to CHW/COPE and the Easy-Care independence score was +0.31 (*p* < 0.001), and with intervals of care assessed by the CHW was +0.25 (*p* < 0.001).

**Table 4:** The validity of continuously distributed COPE assessment scores against clinician judgment (criterion validity - Area under ROC curve) and clinician administered Easy-Care independence score (concurrent validity – Pearson’s correlation)

Impairment	Test	Criterion validity	Concurrent validity
		Area under ROC curve, against clinician judgment	Correlation with EASY CARE independence score
Cognition	Brief CSI-D cognitive score^a^	0.71 (0.61–0.80)	−0.47, *p* < 0.001
	Combined^a^	0.68 (0.58–0.78)	−0.48, *p* < 0.001
Nutrition	MNA—SF score^a^	0.70 (0.62–0.79)	−0.34, *p* < 0.001
Mobility	Gait speed^a^	0.63 (0.45–0.80)	−0.21, *p* = 0.02
	Chair stand^a^	0.65 (0.48–0.83)	−0.38, *p* < 0.001
Depression	GDS-8^b^	0.73 (0.64–0.80)	0.04, *p* = 0.66
Behaviour	NPI severity score^b^	0.72 (0.64–0.80)	0.21,*p* = 0.01
	NPI distress score^b^	0.69 (0.60–0.77)	0.28, *p* = 0.001
Hearing	Best ear^a^	0.71 (0.62–0.80)	−0.14, *p* = 0.10
	Worst ear^a^	0.71 (0.63–0.80)	−0.12, *p* = 0.16

^a^Higher score = less impaired

^b^Higher score = more impaired

### Post hoc analysis

Given the under-identification of impairments by the COPE tool as administered by the CHWs (or, alternatively, the over-identification of impairments by the clinician unstructured assessment), we carried out a post-hoc analysis to compare the EASY-CARE independence scale score among three groups;

1. those who screened positive using the COPE (true positives and false positives),
2. those who were identified with impairment by the clinician, but not by COPE (false negatives),
3. those identified as negative by CHW and clinician (true negatives).

For all impairments other than hearing impairment and depression, those identified by the COPE as impaired (Group 1) had higher independence scale scores (suggesting greater needs for care) than did those identified by the clinician but not confirmed by COPE (Group 2) (Table 5). For nutrition, vision, incontinence and cognitive impairment the difference in mean dependence score between these sub-groups was statistically significant. Finally, in a multivariable model, controlling for age and gender, neither vision nor depression made a statistically significant contribution to EASY-Care independence scores, whether assessed by CHW-administered COPE, or clinician judgment (Table 6). The contribution to the variance made by nutrition, mobility and cognition impairment was greater for CHW/COPE assessed impairment than for clinician judgment, while the reverse was true for impairment in hearing and behaviour.

**Table 5:** Mean EASY-Care independence scores for those identified as impaired by CHW administered COPE assessment (Group 1), compared to those identified as impaired by clinician judgment but not by CHW/COPE (Group 2) and those identified by neither assessor (Group 3)

Impairment	Group 1 CHW COPE + Mean (SD)	Group 2 Clinician +/ CHW COPE-Mean (SD)	Group 3 Both – Mean (SD)	1 vs 3^a^ (mean difference)	1 vs 2^a^ (mean difference)	2 vs 3^a^ (mean difference)
Nutrition	43.8 (21.4)	34.6 (12.3)	26.9 (10.5)	16.9	9.2	7.7
				*p* < 0.001	*p* = 0.04	*p* = 0.20
Mobility	39.3 (19.0)	33.3 (17.8)	23.0 (15.2)	16.3	6.0	10.3
				*p* = 0.16	*p* = 0.76	*p* = 0.53
Vision	40.7 (21.6)	33.1 (13.0)	Not estimated^b^	Not estimated^b^	7.6	Not estimated^b^
					*p* = 0.02	
Hearing	36.3 (15.4)	40.3 (24.2)	29.9 (2.5)	6.4	−3.9	10.4
				*p* = 0.21	*p* = 0.68	*p* = 0.15
Continence	56.5 (25.5)	43.7 (15.2)	29.1 (9.4)	27.4	12.8	14.6
				*p* < 0.001	*p* = 0.006	*p* < 0.001
Cognition	48.8 (22.0)	32.1 (13.0)	28.0 (11.8)	20.8	16.6	4.1
				*p* < 0.001	<0.001	*p* = 0.58
Mood	38.4 (19.6)	39.5 (19.4)	30.6 (11.4)	7.9	−1.1	8.9
				*p* = 0.29	*p* = 0.95	*p* = 0.26
Behaviour	42.6 (22.1)	39.1 (17.8)	29.6 (9.6)	12.9	3.5	9.4
				*p* = 0.01	*p* = 0.66	*p* = 0.08

^a^Scheffe test for mean difference with multiple sub-group comparisons

^b^Could not be computed as only one participant in this sub-group

**Table 6:** Independent, individual and collective contribution of impairments ascertained through CHW administered COPE and clinician judgment to the variance (eta squared %) in EASY-Care independence score

Impairment	Mean difference (95 % confidence intervals) and variance explained (eta^2^ %)	
	CHW COPE assessment	Clinician judgment
Nutrition	−6.3 (−11.4 to −1.2) 4.5 %	−7.1 (−13.1 to −1.1) 3.8 %
Mobility	−7.8 (−14.2 to −1.4) 4.3 %	−12.6 (−23.0 to −2.1) 3.9 %
Hearing	−0.8 (−6.6 to 5.0) 0.1 %	−5.9 (−11.7 to 0.0) 2.8 %
Vision	−0.6 (−5.6 to 4.4) (0.4 %)	22.0 (−3.9 to +47.8) (2.0 %)
Mood	1.2 (−3.9 to 6.4) (0.2 %)	+0.1 (−8.1 to +8.3) (0.0 %)
Behavior	−2.7 (−7.8 to 2.5) 0.8 %	−12.1 (−18.5 to −5.7) 9.2 %
Cognition	−9.6 (−15.3 to −4.0) 8.2 %	−1.9 (−3.5 to +4.9) 0.0 %
Total	17.8 %	19.7 %

### Qualitative data on COPE assessment: community health workers (CHWs)

The COPE assessment package was generally perceived as easy to administer, with CHWs reporting that they gained knowledge, experience and confidence through training. The CHWs felt empowered to conduct assessments of older people, and discriminate between different impairments that might require intervention. In their view this could increase the efficiency of the care provided, both in improving identification and generating more accurate referrals. However, several of the CHWs thought that the involvement of doctors was crucial, to validate their findings, to recommend and implement treatment, and ensure adherence. The assessment was generally perceived as acceptable to the older people and their family members, in part because this showed that the service was interested in their problems. Benefit might come simply from improved knowledge and understanding. However, some CHWs did find it difficult to convince some older people of the benefits of assessment, given their fatalistic view of their health status. Opinion was divided among CHWs as to whether the COPE assessment could be routinely incorporated in their daily work. Some felt that it was both feasible and necessary. Others expressed concern about the time to administer the COPE assessment, and the impact that this might have on their other work. However, four CHWs volunteered that they had already begun to, or intended to use the COPE in their clinical practice.

The main difficulties experienced in using the COPE tool were that planning was required to carry out the caregiver assessment, and sometimes a second visit was required for that purpose. Some tests, particularly the 5 m walk test and the visual acuity test were difficult to perform in some households because of cramped space and/or poor lighting. The visual acuity test was difficult to explain to participants with cognitive impairment, and some health workers expressed a need for additional training to identify vision problems in older people with dementia. Some CHWs commented that a second assistant might be required with very frail older people when no family caregiver was at hand (for detail information see the Additional file 1).

## Discussion

The first objective of this study was to examine the acceptability and utility of comprehensive COPE assessment developed for non-specialist community health workers in identifying specific impairments in frail older people at primary health care level. Our second objective was to explore the concurrent validity of COPE assessment against clinical assessments carried out by physicians working in the same local public health system.

The strengths of this study included, first, a clear objective to develop a comprehensive geriatric assessment tool for use in resource poor settings by suitably trained CHWs, with little or no assumed relevant clinical experience or knowledge. The pragmatic study design assessed how the structured assessment might work in real-world primary care settings, and how the results of the assessment might converge with those of clinicians working in the same settings. The clinicians conducted an independent assessment and were completely masked from the CHW COPE assessment results. The clinician assessment may have been adversely affected by the doctors’ non-specialist background, the short time available for the assessment, and the lack of equipment (for, for example audiometry or visual acuity testing). For all these reasons, the clinician judgment certainly cannot be considered to represent a ‘gold standard’. We have carried out a construct validation rather than a criterion validation of the COPE assessment. It would be possible, perhaps desirable, to carry out a more detailed criterion validation of the COPE in the future. However, evidence, mainly from high income countries, already supports criterion validity for most of the components. Arguably, the convergence with local clinician opinion may be most relevant to considering its utility and acceptability within the local health system.

There was only a moderate agreement between the CHW COPE assessment and the clinician judgment for some of the impairments; nutrition, continence, mood, hearing and behaviour; and low agreement for mobility, cognition and vision impairments. On closer inspection of the data, this was mainly accounted for by the generally higher prevalence of all of the impairments other than hearing impairment, according to clinician judgment compared with the findings from the structured COPE assessment. Impairments identified by COPE were generally confirmed by the clinicians, reflected in the high positive predictive values for the COPE assessment (72.2 to 98.5 %). However, particularly for vision, cognition and mood impairment, many more participants were considered by the clinician to have the impairment, reflected in the large discrepancy in prevalence, the low sensitivity of the COPE assessment, and the low levels of agreement. Since we lack an independent gold standard assessment, it is impossible to be sure whether this represents under-recognition by the COPE, or over-diagnosis by the clinician assessor, or both. In the 10/66 Dementia Research Group’s population-based surveys in Latin America, India and China, the prevalence of dementia among care dependent participants was around 50 % in most sites [62]. This is closer to the COPE estimate of 38 % with cognitive impairment than the clinician estimate of 78 %. There are no suitable external comparators for the prevalence of low mood and visual impairment among care dependent older people. The level of disability/ needs for care among those identified by COPE was generally higher than that for those additionally identified by the clinicians but not confirmed by COPE. This difference was both particularly striking and statistically significant for nutrition, cognition and vision impairments, suggesting that COPE might be more conservative than clinician judgment and more effective at targeting those with more severe impairment. This may be because the CHW COPE assessment comprised objective tests with clear operationalisation, whereas the clinicians relied upon global clinical impression.

Reassuringly, COPE assessed impairments were generally associated with EASY-Care independence scores. The lack of any crude or adjusted association between mood impairment and disability, whether mood was assessed by COPE or clinician judgment, is surprising. It may be that in this sample of older people with extensive multimorbidity and intensive needs for care, the impact of other conditions and impairments predominates. Whether assessed by COPE or clinician judgment, impairments in nutrition, mobility, hearing, vision, mood, behaviour and cognition collectively accounted for just under 20 % of the variance in the independence score, with the largest contributions coming from cognition/behaviour, consistent with other reports of the dominant effect of disorders of the brain and mind on disability and dependence [63, 64].

Most CHWs reported that the COPE assessment was relatively easy to administer, and appreciated the empowerment that the training and tool gave them to conduct competent assessments of older people and identify specific problems. Benefits cited included the potential to increase the coverage of care for older people, improve the efficiency of services, and optimise family care arrangements. Some CHWs talked of this as a necessary service development and expressed willingness to use the COPE assessment in the future. However, there were concerns regarding the time taken to administer the assessment, and its impact on their current workload. These views are likely coloured by the fact that attending to the needs of older people is currently neither part of their role, nor on the priority healthcare agenda of the primary health care or sub-centre system [12]. Therefore, spending time with older people is considered to be an additional responsibility.

Some needs for refinement were noted. A portable light source and a mirror could facilitate the administration of the Snellen chart vision test, where space is cramped and lighting inadequate. Vision testing should be extended to include near vision (reading), since unlike refraction errors (which would require optometry and a prescription for glasses or cataract surgery), hyperopia can be corrected by low cost magnifying lens glasses, which could be dispensed by the CHW. Pain is a common and burdensome impairment, [28] which is not yet assessed as part of COPE, and may be a relevant co-factor in other impairments. Pain management may be challenging unless prescribing restrictions that pertain in many health systems, including the Goan system, are eased. More information would be required to plan interventions; for example a dietary assessment for nutritional intervention, and assessment of pain, recent fractures, and safety aspects prior to exercise interventions to improve mobility. However, such additional assessments could be conducted as part of the intervention, after screening using COPE.

## Conclusion

The evidence presented here suggests that the COPE assessment is a useful tool for identifying specific impairments linked to needs for home care and support. In low-resourced primary health care, it is rare for clinicians to visit patients in the community, and this is even considered undesirable [9]. Mobility impairment and lack of transportation limits the scope for frail dependent older people to visit the primary health care facilities for assessment and treatment. Community health workers (who are currently the interface between the community and primary health care facility) could bridge this gap. The high positive predictive value of the CHW identification using the COPE assessment tool suggests that local physicians could have confidence in the accuracy of the CHW assessments, whether in authorising them to initiate home-based interventions, or in accepting referrals arising from these assessments. This collaborative working model is already used to improve maternal and child health, but its potential is rarely considered for managing dependent older people.

The next step would be to evaluate the COPE for cost–effectiveness, in the context of a cluster randomised controlled trial of a complex intervention comprising screening and intervention with evidence-based packages of care. Such multidimensional community-based assessments have been shown to be effective in maintaining physical function, reducing falls and hospitalization in high income countries, although their incremental benefit was less apparent in trials conducted in more recent years, when these approaches became more widespread, and access to basic healthcare was more assure [65].

## Additional file

Additional file 1:
**Qualitative data analysis.** Community Health Workers (CHWs) experiences and attitudes regarding the use of the COPE assessment. (DOCX 16 kb)

## Abbreviations

CHW: Community Health Worker
LAMICs: Low And Middle Income Countries
WHO: World Health Organization
MNA: Mini Nutritional Assessment
BMI: Body Mass Index
CSI-D: Community Screening Instrument for Dementia
DSM-IV: Diagnostic and Statistical Manual of Mental Disorders, 4th edition
GDS: Geriatric Depression Scale
IADL: Instrumental Activities of Daily Living
ADL: Activities of Daily Living

## References

[1] Vos T, Flaxman AD, Naghavi M, Lozano R, Michaud C, Ezzati M (2012). Years lived with disability (YLDs) for 1160 sequelae of 289 diseases and injuries 1990–2010: a systematic analysis for the Global Burden of Disease Study 2010. Lancet.

[2] Wea H (2013). Age-specific and sex-specific mortality in 187 countries, 1970–2010: a systematic analysis for the Global Burden of Disease Study 2010. Lancet.

[3] Spiers NA, Matthews RJ, Jagger C, Matthews FE, Boult C, Robinson TG (2005). Diseases and Impairments as Risk Factors for Onset of Disability in the Older Population in England and Wales: Findings From the Medical Research Council Cognitive Function and Ageing Study. J Gerontol A Biol Sci Med Sci.

[4] Stuck AE, Walthert JM, Nikolaus T, Bula CJ, Hohmann C, Beck JC (1999). Risk factors for functional status decline in community-living elderly people: a systematic literature review. Soc Sci Med.

[5] Smith SM, Soubhi H, Fortin M, Hudon C, O'Dowd T (2012). Interventions for improving outcomes in patients with multimorbidity in primary care and community settings. Cochrane Database Systematic Review.

[6] Albanese E, Liu Z, Acosta D, Guerra M, Huang Y, Jacob KS (2011). Equity in the delivery of community healthcare to older people: findings from 10/66 Dementia Research Group cross-sectional surveys in Latin America, China, India and Nigeria. BMC Health Serv Res.

[7] Beaglehole R, Epping-Jordan J, Patel V, Chopra M, Ebrahim S, Kidd M (2008). Improving the prevention and management of chronic disease in low-income and middle-income countries: a priority for primary health care. Lancet.

[8] Samb B, Desai N, Nishtar S, Mendis S, Bekedam H, Wright A (2010). Prevention and management of chronic disease: a litmus test for health-systems strengthening in low-income and middle-income countries. Lancet.

[9] IPHS (2012). Indian Public Health Standards(IPHS) Guidelines for Primary Health Centres In.

[10] Roy S (1985). Primary Health Care in India. Health and Population-Perspectives & Issues.

[11] Malik G (2009). Role of auxiliary nurse midwives in National Rural Health Mission. Nurs J India.

[12] GoI. Indian Public Health Standards(IPHS), Guidelines for Sub-Centres Revised In*.* Edited by Directorate General of Health Services MoHFW. New Delhi, India: Government of India(GoI); 2012.

[13] Jotheeswaran AT (2014). Identification, Prevention, and Management of Dependency Among Frail Dependent Older People in Low Resourced Primary Health Care Settings.

[14] Huss A, Stuck AE, Rubenstein LZ, Egger M, Clough-Gorr KM (2008). Multidimensional preventive home visit programs for community-dwelling older adults: a systematic review and meta-analysis of randomized controlled trials. J Gerontol A Biol Sci Med Sci.

[15] Stuck AE, Siu AL, Wieland GD, Rubenstein LZ, Adams J (1993). Comprehensive geriatric assessment: a meta-analysis of controlled trials. Lancet.

[16] Li C-M, Chen C-Y, Li C-Y, Wang W-D, Wu S-C (2010). The effectiveness of a comprehensive geriatric assessment intervention program for frailty in community-dwelling older people: a randomized, controlled trial. Arch Gerontol Geriatr.

[17] Guidance on Frailty [http://www.who.int/ageing/health-systems/integrated-care/en/] Accessed 11 Oct 2015.

[18] Philp I, Lowles RV, Armstrong GK, Whitehead C (2002). Repeatability of standardized tests of functional impairment and well-being in older people in a rehabilitation setting. Disabil Rehabil.

[19] Fillenbaum GG, Smyer MA (1981). The development, validity, and reliability of the OARS multidimensional functional assessment questionnaire. J Gerontol.

[20] Lennon OC, Carey A, Creed A, Durcan S, Blake C (2011). Reliability and validity of COOP/WONCA functional health status charts for stroke patients in primary care. J Stroke Cerebrovasc Dis.

[21] Mann E, Koller M, Mann C, van der Cammen T, Steurer J (2004). Comprehensive Geriatric Assessment (CGA) in general practice: results from a pilot study in Vorarlberg. Austria BMC Geriatrics.

[22] EASY-Care Standard [http://www.easycareproject.org/the-easycare-instruments.html] Accessed 10 Oct 2015.

[23] AbellanvanKan G, Rolland Y, Andrieu S, Bauer J, Beauchet O, Bonnefoy M (2009). Gait speed at usual pace as a predictor of adverse outcomes in community-dwelling older people an International Academy on Nutrition and Aging (IANA) Task Force. The Journal of Nutrition, Health & Aging.

[24] Gobbens RJ, Luijkx KG, Wijnen-Sponselee MT, Schols JM (2010). Towards an integral conceptual model of frailty. J Nutr Health Aging.

[25] Peel NM, Kuys SS, Klein K (2013). Gait speed as a measure in geriatric assessment in clinical settings: a systematic review. J Gerontol A Biol Sci Med Sci.

[26] Mijnarends DM, Meijers JM, Halfens RJ, ter Borg S, Luiking YC, Verlaan S (2013). Validity and reliability of tools to measure muscle mass, strength, and physical performance in community-dwelling older people: a systematic review. J Am Med Dir Assoc.

[27] Prince M, Ferri C, Acosta D, Albanese E, Arizaga R, Dewey M (2007). The protocols for the 10/66 dementia research group population-based research programme. BMC Public Health.

[28] Prince M, Acosta D, Ferri CP, Guerra M, Huang Y, Jacob KS (2011). The association between common physical impairments and dementia in low and middle income countries, and among people with dementia, their association with cognitive function and disability. A 10/66 Dementia Research Group population-based study. Int J Geriatr Psychiatry.

[29] Barbosa AR, Souza JM, Lebrao ML, Laurenti R, Marucci Mde F (2005). Functional limitations of Brazilian elderly by age and gender differences: data from SABE Survey. Cadernos De Saude Publica.

[30] Jones CJRR (1999). Functional fitness normative scores for community residing older adults ages 60–94. J Aging Phys Act.

[31] Kawabata Y, Hiura M (2008). The CS-30 test is a useful assessment tool for predicting falls in commuity-dwelling elderly people. Rigakuryoho Kagaku.

[32] Rubenstein LZ, Harker JO, Salva A, Guigoz Y, Vellas B (2001). Screening for undernutrition in geriatric practice: developing the short-form mini-nutritional assessment (MNA-SF). J Gerontol A Biol Sci Med Sci.

[33] Cai-hua ZHANG (2010). Malnutrition screening by Mini-Nutritional Assessment and Short-Form Mini-Nutritional Assessment in patients with Alzheimer's disease. Chinese Journal of Clinical Nutrition.

[34] Kaiser MJ, Bauer JM, Ramsch C, Uter W, Guigoz Y, Cederholm T (2009). Validation of the Mini Nutritional Assessment short-form (MNA-SF): a practical tool for identification of nutritional status. J Nutr Health Aging.

[35] Tsai AC, Chang T-L, Wang Y-C, Liao C-Y (2010). Population-Specific Short-Form Mini Nutritional Assessment with Body Mass Index or Calf Circumference Can Predict Risk of Malnutrition in Community-Living or Institutionalized Elderly People in Taiwan. J Am Diet Assoc.

[36] Marmamula S, Narsaiah S, Shekhar K, Khanna RC, Rao GN (2013). Visual Impairment in the South Indian State of Andhra Pradesh: Andhra Pradesh - Rapid Assessment of Visual Impairment (AP-RAVI) Project. PLoS One.

[37] Keeffe JE, Lovie-Kitchin JE, Maclean H, Taylor HR (1996). A simplified screening test for identifying people with low vision in developing countries. Bull World Health Organ.

[38] Hsu W-M, Cheng C-Y, Liu J-H, Tsai S-Y, Chou P (2004). Prevalence and causes of visual impairment in an elderly Chinese population in Taiwan: The Shihpai Eye Study. Ophthalmology.

[39] Mbulaiteye SM, Reeves BC, Karabalinde A, Ruberantwari A, Mulwanyi F, Whitworth JA (2002). Evaluation of E-optotypes as a screening test and the prevalence and causes of visual loss in a rural population in SW Uganda. Ophthalmic Epidemiol.

[40] WHO (2010). International Statistical Classification of Diseases and Related Health Problems. Vol. 1. 10th. rev.

[41] Bagai A, Thavendiranathan P, Detsky AS (2006). Does this patient have hearing impairment?. The Journal of American Medical Association.

[42] Swan IR (1985). The whispered voice as a screening test for hearing impairment. J R Coll Gen Pract.

[43] Macphee GJ, Crowther JA, McAlpine CH (1988). A simple screening test for hearing impairment in elderly patients. Age Ageing.

[44] Pirozzo S, Papinczak T, Glasziou P (2003). Whispered voice test for screening for hearing impairment in adults and children: systematic review. Br Med J.

[45] McShefferty D, Whitmer WM, Swan IR, Akeroyd MA: The effect of experience on the sensitivity and specificity of the whispered voice test: a diagnostic accuracy study. British Medical Journal Open 2013, 3(4): doi:10.1136/bmjopen-2012-002394. 10.1136/bmjopen-2012-002394 PMC3641455 23604349

[46] Prince M, Acosta D, Chiu H, Scazufca M, Varghese M (2003). Dementia diagnosis in developing countries: a cross-cultural validation study. Lancet.

[47] Prince M, Acosta D, Ferri CP, Guerra M, Huang Y, Jacob KS (2011). A brief dementia screener suitable for use by non-specialists in resource poor settings the cross-cultural derivation and validation of the brief Community Screening Instrument for Dementia. International Journal of Geriatric Psychiatry.

[48] Yesavage JA, Brink TL, Rose TL, Lum O, Huang V, Adey M (1982). Development and validation of a geriatric depression screening scale: a preliminary report. J Psychiatr Res.

[49] Kohli A, Banerjee ST, Verma SK (1991). Adaptation of a Geriatric Depression Scale in simple Hindi. Indian Journal of Clinical Psychology.

[50] Jongenelis K, Gerritsen DL, Pot AM, Beekman AT, Eisses AM, Kluiter H (2007). Construction and validation of a patient- and user-friendly nursing home version of the Geriatric Depression Scale. Int J Geriatr Psychiatry.

[51] Kaufer DI, Cummings JL, Ketchel P, Smith V, MacMillan A, Shelley T (2000). Validation of the NPI-Q, a brief clinical form of the Neuropsychiatric Inventory. J Neuropsychiatry Clin Neurosci.

[52] Boada M, Cejudo JC, Tarraga L, Lopez OL, Kaufer D (2002). [Neuropsychiatric inventory questionnaire (NPI-Q): Spanish validation of an abridged form of the Neuropsychiatric Inventory (NPI)]. Neurologia.

[53] Philp I (1997). Can a medical and social assessment be combined?. J R Soc Med.

[54] Mahoney FI, Barthel DW (1965). Functional Evaluation: The Barthel Index. Md State Med J.

[55] Brazier JE, Harper R, Jones NM, O'Cathain A, Thomas KJ, Usherwood T (1992). Validating the SF-36 health survey questionnaire: new outcome measure for primary care. Br Med J.

[56] Katzman R, Brown T, Fuld P, Peck A, Schechter R, Schimmel H (1983). Validation of a short Orientation-Memory-Concentration Test of cognitive impairment. Am J Psychiatry.

[57] D'Ath P, Katona P, Mullan E, Evans S, Katona C (1994). Screening, detection and management of depression in elderly primary care attenders. Fam Pract.

[58] Heikinnen E, Waters WE, Brzeziński ZJ (1983). The Elderly in eleven countries: a sociomedical survey.

[59] Bath P, Philp I, Boydell L, McCormick W, Bray J, Roberts H (2000). Standardized health check data from community-dwelling elderly people: the potential for comparing populations and estimating need. Health Soc Care Community.

[60] Döhner HKC, Philp I (1999). Health outcome instrumente in der primärversonrgung älterer menschen erste ergebnisse einer konzertierten action de Europe (SCOPE). Geriatrie Forschung.

[61] Wojszel ZBBB, Politynska B (1999). Ocena stanur funkcjonowania ludzi w podeslym wieku przez lekarza rodzinnego za pomoca kwestionariusza EasyCare. Polski Merkuriusz.

[62] WAR (2013). World Alzheimer Report 2013, Journey of Caring: An analysis of long-term care for dementia.

[63] Sousa RM, Ferri CP, Acosta D, Albanese E, Guerra M, Huang Y (2009). Contribution of chronic diseases to disability in elderly people in countries with low and middle incomes: a 10/66 Dementia Research Group population-based survey. Lancet.

[64] Sousa RM, Ferri CP, Acosta D, Guerra M, Huang Y, Jacob K (2010). The contribution of chronic diseases to the prevalence of dependence among older people in Latin America, China and India: a 10/66 Dementia Research Group population-based survey. BMC Geriatr.

[65] Beswick AD, Rees K, Dieppe P, Ayis S, Gooberman-Hill R, Horwood J (2008). Complex interventions to improve physical function and maintain independent living in elderly people: a systematic review and meta-analysis. Lancet.

