# Supplementary material for: Identifying common impairments in frail and dependent older people: validation of the COPE assessment for non-specialised health workers in low resource primary health care settings
Source: BMC Geriatr. 2015 Oct 14;15:123. doi: 10.1186/s12877-015-0121-1 (PMC4607017; doi:10.1186/s12877-015-0121-1)
Supplement: Additional file 1: — Qualitative data analysis. Community Health Workers (CHWs) experiences and attitudes regarding the use of the COPE assessment. (DOCX 16 kb) [file 12877_2015_121_MOESM1_ESM.docx]

Qualitative data analysis

Community Health Workers (CHWs) experiences and attitudes regarding the use of the COPE assessment

The COPE assessment package was generally perceived as easy to administer, with CHWs reporting that they gained knowledge, experience and confidence through training.

“COPE assessment was designed in a very simple way; therefore we had no difficulty in using it. We had used it at ground level and it wasn't expensive also. Tomorrow if anyone require assessment, most of the things are ready to administer”. Respondent - A

“It was not easy in the beginning. You came with us and taught us properly. We also came to know about our mistakes. After that it was easy with the rest of the patients”. Respondent- B.

The CHWs felt empowered to conduct assessments of older people, and discriminate between different impairments that might require intervention

“We got to learn a lot of new thing from it, which we were not doing in our routine work. Mostly we refer older patients to the doctors. But in this we were playing the role of a doctor and assessing older patient, it was interesting and we were curious to identify their problems”. Respondent - D.

“The COPE tool was very easy and we were doing it as if we were the doctor. After administering the test at home we came to know what exactly their problems is like their vision power, can or cannot hear or to what extent they are depressed or cannot remember, or having incontinence”. Respondent - E

“We are able to distinguish between their problems. Sometimes what happens, older people experience psychological problem and they don’t eat well and may be become undernourished. In such cases, we could not understand whether it was psychological or nutrition problem? But now we can separate the nutrition and psychological problems, and refer them as per their need”. Respondent – F.

In their view this could increase the efficiency of the care provided, both in improving identification and generating more accurate referrals

“The skills have developed and now the job is also getting done in a better way. Therefore I am interested. Earlier if anyone was telling us that they cannot see we simply ask them to go to the PHC (Primary Health Centre). Now we feel good to look at their problem, we tell them let us go inside (home) and do the test”. Respondent - B.

“If the tools were not there we would refer them to the doctor directly and we would not be able to tell their exact problem to them and their family. Now if they tell us that they cannot see or hear or they cannot remember or have incontinence - we can do these tests with them and tell them and their family that these specific problems. Moreover, according to the test results we can refer them for hearing and vision”. Respondent - E

“But now, since we know we have the training and assessment tool in our hand, we can use them and assess the older person. Even if nothing could be done at our level, we could at least refer them to appropriate service. In this regard the COPE assessment tool was useful to find out what exactly the problem is and then refer them accordingly”. Respondent - A

“Only when we identify the real problems of older people, we would be able to refer them to the specialist doctor. In case if we don’t have any idea about their problems, it not easy to refer them”. Respondent -F

“We see a group in whom even the conditions that are treatable were not treated at home. Mainly because assessments were not done properly” Respondent - A

After knowing his/her history, we can tell the doctor about older person problems. Because when they go to the OPD, the doctor does not have so much time to assess everything. So sometimes it is neglected because the doctor is busy”. Respondent - F

However, several of the CHWs thought that the involvement of doctors was crucial, to validate their findings, to recommend and implement treatment and ensure adherence.

“We only do the identification, but the treatment part which is very important. We cannot give treatment. Finally, for any treatment you need the doctor, therefore doctors visit is important”. Respondent - F

“The doctors should assess them and give treatment or solution to the identified problems. Otherwise there is no benefit from our assessment”. Respondent - B

“Yes, I feel so. Because at times we may be making some mistake. Until the doctor confirm, the older patients do not get agree 100 percent”. Respondent - G

“We used to do this whisper voice test, some older people complain about pain in their ears or some other problems for which doctor is needed”. Respondent -A

The assessment was generally perceived as acceptable to the older people and their family members, in part because this showed that the service was interested in their problems. Benefit might come simply from improved knowledge and understanding. However, some CHWs did find it difficult to convince some older people of the benefits of assessment, given their fatalistic view of their health status.

“They really felt that we will get some information about their sickness through assessment. And they came to know what difficulties and problems they had, which satisfied them”. Respondent - H

“Somebody was concerned about their health. So older people and as well as the family members were genuinely showed good interest”. Respondent- A

“Most of the family members, as well as, older people who were not able to perform their daily activities were willingly participated. Because they knew that something is been assessed and they would benefit by at least knowing what are their problems”. Respondent – A

“Most of the older patients were agreed for assessment and okay with it. But few directly say “No, we don’t require, what are you going to do? We are like this for so many years. You just come for one day, there is not going to be any change at all”. Respondent - A

Opinion was divided among CHWs as to whether the COPE assessment could be routinely incorporated in their daily work. Some felt that it was both feasible and necessary. Others expressed concern about the time to administer the COPE assessment, and the impact that this might have on their other work. However, four CHWs volunteered that they had already begun to, or intended to use the COPE in their clinical practice.

“We did not have any concerns about the time. In the beginning we felt that this is going to take a lot of time and we have our other targets too. But later in the field it became a usual thing and perfect for us, so there was no big problem - It was easy to use it”. Respondent - E

“There was no problem about the time. Whatever required has to be done and should be done correctly. For that reason we would take whatever time that was required. So that we get the correct answer from older patients and family members”. Respondent - D

“Not that easy I would say. Tools were very good - they are simple to be understood by the old people. But then administration taking bit longer time. It be may be because, older people have difficulties in hearing, walking, and their age. So it wasn’t that easy to administrate I would say. Generally, other works takes lesser time - suppose we attend to two to three older people then most of our time for the day will be consumed”. Respondent -A

“We had to take a little trouble since it was extra work. Mainly because it required 30 to 45 minutes with one older patient, and to complete the work we had to go early to the field and work till lunch time”. Respondent - E

“Only thing - we have heavy work load. Nowadays, time available to do the work is not sufficient. Therefore additional work is impossible. We only have morning hours for field visits; we have to complete all our work with that time”. Respondent - C

“We use it in our practice and we can also use it in future. Whenever we go to houses, we will use it”. Respondent - B

“I am ready to do the administration because I did not have any problem. All older people welcomed me because they will get some benefit this work. So I am ready to it in the future”. Respondent - H

The main difficulties experienced in using the COPE tool were that organisation was required to carry out the caregiver assessment, and sometimes a second visit was required for that purpose. Some tests, particularly the five metre walk test and the visual acuity test were difficult to perform in some households because of cramped space and/ or poor lighting. The visual acuity test was difficult to explain to participants with cognitive impairment, and some health workers expressed a need for additional training to identify vision problems in older people with dementia. Some CHWs commented that a second assistant might be required with very frail older people when no family caregiver was at hand.
